# Quality is our lifeblood

**DOI:** 10.1093/jhps/hnu016

**Published:** 2014-11-11

**Authors:** Richard Villar

**Affiliations:** Editor-in-Chief

I have had the privilege of editing for many years and for a variety of different journals. But then along comes *JHPS*, the *Journal of Hip Preservation Surgery*, our journal, your journal, something different, something new. Issue 1 is now out there and, if the welcoming comments we received at the recent International Society for Hip Arthroscopy meeting in Rio de Janeiro are anything to go by, *JHPS* is already filling a much-needed gap.

To develop a new journal clearly takes time and when that baby takes its first steps, as an Editor-in-Chief you bite your nails to the quick. At least I do. I now have these stubby, fleshy things which might pass for fingers but having seen the quality of the first issue I realize I need not have worried. The quality is clear, the support has been tremendous, and the willingness of authors to submit and to write with such enthusiasm is something that I thought had escaped me in previous journals. And you should see the reviews. They are brilliant, fantastic, in a league of their own. Now, when you submit your paper to *JHPS* you will know that an expert in the field will review it. At long last, someone will see your paper for the specialist beast it is and someone, admittedly anonymized, will give your labours a fair hearing. Reviewers, of course, are the unsung heroes of any peer-reviewed journal. They are the guardians of quality; without them a journal such as *JHPS* simply could not exist.

Unsurprisingly, reviewers do not always agree. Indeed it is quite common that they take different, almost polarized views to the same paper. ‘Accept!’ clamours one, ‘Revise!’ yells another and ‘Reject!’ shouts the third, should we seek a further opinion. Yet although a reviewer’s conclusion is important, more critical is the analysis he (or she) chose to reach a decision. That seems to be where *JHPS* presently excels. The reviews are detailed, well constructed, in no way vindictive or biased and frequently immensely supportive. After all, a reviewer’s job is not just to decide whether a submission should be published but also to offer advice as to how it might be improved so that it can be accepted next time round. I call such submissions ‘almost-papers’. They are nearly there, not quite, but almost. A little jig here, a tiny jog there, a revamp of a discussion and a paper that might be rejected by journals that are not blessed with specialist reviewers, instantly becomes acceptable to *JHPS*. I simply had not realized how valuable specialist reviewers can be. They are, in my view, a breed apart and utter gold dust.

Yet although *JHPS* can hold its head up high, all readers and authors will be aware of potential flaws in the wider biomedical review system. What we have with our journal is not always available elsewhere. It can, for example, sometimes be dangerous to ask authors for their own recommendation of suitable reviewers, as it is not unknown for a recommendation to be falsified. Fabricated identities and contact details are proffered so that the peer review request goes straight back to the originating author. How simple it is for that author to give his own submission a perfect bill of health. Don’t believe me? Have a look at the *New York Times* [1] or the statement by SAGE [2] whose publication the *Journal of Vibration and Control* was unfortunately affected by this. Sixty papers were retracted as a consequence.

Many journals have great difficulty in finding suitably qualified reviewers for specialist papers. This is a particular problem for the more general publications. One survey of peer review suggested that 56% of reviewers felt there was lack of guidance on how to review while 68% thought that formal training might be of value [3]. That said, training schemes have not always been the panacea sought [4] and performance has even declined over time [5]. As Patel states in an excellent paper on this topic [6], it may be that by the time a researcher has reached the stage in their career when they start to peer review, it is too late to teach peer review anyway.

So thank Heavens for the *JHPS* reviewer team and our excellent Editorial Board. They are doing well and, as Editor-in-Chief, I trust them implicitly. They are most certainly our guardians of high standard. For those who have read our inaugural issue, I trust you agree that quality was up to par. I particularly enjoyed O’Donnell *et al.* [7] and their comprehensive review of the ligamentum teres, but then those who know me would not be surprised at this. It is actually inappropriate that I should select a favourite paper anyway, as all the papers in that first issue were good. Be aware that sadly a fair few submissions to that first issue did not make it into print; roughly 40% were rejected, including one of my own.

And this, our second issue, the second in what feels such a very short time? Quality is once again within its core. Why not start by looking at Hogervorst and Vereecke’s review on the evolution of the osseous human hip [8]? I found this fascinating and look forward to their part 2 on the evolution of the hip’s soft tissues that will appear in the issue after this. Yet there are plenty of other papers, too, each with a clear message of its own and each a publication you should not miss. Please read, savour and enjoy. After all, *JHPS* is for you.

My very best wishes to you all.
